# What should we do to optimise outcome in twin pregnancy complicated with placenta percreta? A case report

**DOI:** 10.1186/s12884-015-0714-x

**Published:** 2015-11-05

**Authors:** Mehmet Aral Atalay, Fatma Oz Atalay, Bilge Cetinkaya Demir

**Affiliations:** Department of Obstetrics and Gynecology, Uludag University School of Medicine, Bursa, Turkey; Department of Surgical Pathology, Uludag University School of Medicine, Bursa, Turkey; Uludağ Üniversitesi Tıp Fakültesi Kadın Hastalıkları ve Doğum Anabilim Dalı, Görükle, 16059 Bursa Turkey

**Keywords:** Hemorrhage, Infection, Morbidly adherent placenta, Placenta percreta, Twin pregnancy

## Abstract

**Background:**

Patients with morbidly adherent placenta (MAP) are under risk of massive bleeding. It readily necessitates very complicated surgery and massive blood transfusion, and even leads to mortality. Cesarean hysterectomy (CH) is the procedure that is acknowledged worldwide, since it helps to minimize complications.

**Case presentation:**

A patient with dichorionic twin pregnancy underwent to cesarean section (CS) due to preliminary diagnosis of placenta percreta at her 35^th^ week of pregnancy. Both of the placentas were left in situ. The patient admitted with signs of infection. Emergency total abdominal hysterectomy was performed 7 weeks after CS. In the course of hysterectomy, 3 units of erythrocyte suspension and 2 units of fresh frozen plasma were transferred, whereas none was required during CS.

**Conclusion:**

Abandoning placenta in situ seems to be a logical alternative to the CH in patients with placenta percreta in order to minimize complications related to massive blood transfusion and surgical technique. However, it appears to increase maternal morbidity due to maternal infection in twin pregnancy.

## Background

Abnormal placental invasion, which is also called as morbidly adherent placenta (MAP), is considered as one of the most severe complications of pregnancy [1]. MAP is a potential life-threatening condition. Patients with MAP are under risk of massive bleeding due to spontaneous or forced separation of the placenta. Therefore, cesarean hysterectomy (CH) is the procedure that is acknowledged worldwide to prevent such complications in patients with diagnosis of MAP. However, Sentilhes et al. have tried an alternative approach and demonstrated that uterine conservation is possible in patients with MAP [1]. In this report, we present a case of MAP in a dichorionic (DC) twin pregnancy who is followed up with the retained placentas. This is the first reported case of a DC twin pregnancy in which both of the placentas were MAPs and were left in situ during cesarean section (CS). We also discuss the advantages and disadvantages of abandoning placenta in situ in such situations.

## Case presentation

### Patient

Thirty-three years old women with dichorionic diamniotic twin pregnancy admitted to our perinatology clinic at her 28^th^ gestational week with a preliminary diagnosis of complete placenta previa. She had two healthy-living children, one of which was delivered by CS, and one spontaneous abortion, which ended up with curettage.

**Fig. 1:**
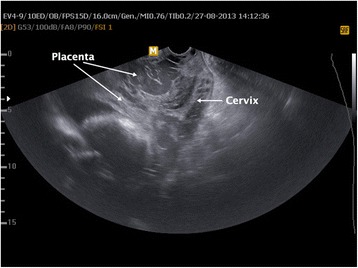
Antepartum transvaginal ultrasonographic survey. Note the tortuous vessels anterior to the lower uterine segment just overneath the cervix

Follow-up of the patient was done weekly until 35^th^ gestational age. Prior to the delivery, we were unable to determine the myometrial thickness at uterovesical contiguity by ultrasonography. Additionally, placental lacunes were prominent, and there were multiple tortuous vessels at uterovesical junction (Fig. 1). Preliminary diagnosis was placenta percreta. Two-step surgery, first remaining the placenta in utero with intention of afterward-hysterotomy and -metroplasty, was planned to decrease complications due to surgery and massive blood transfusion.

### Procedures

**Fig. 2:**
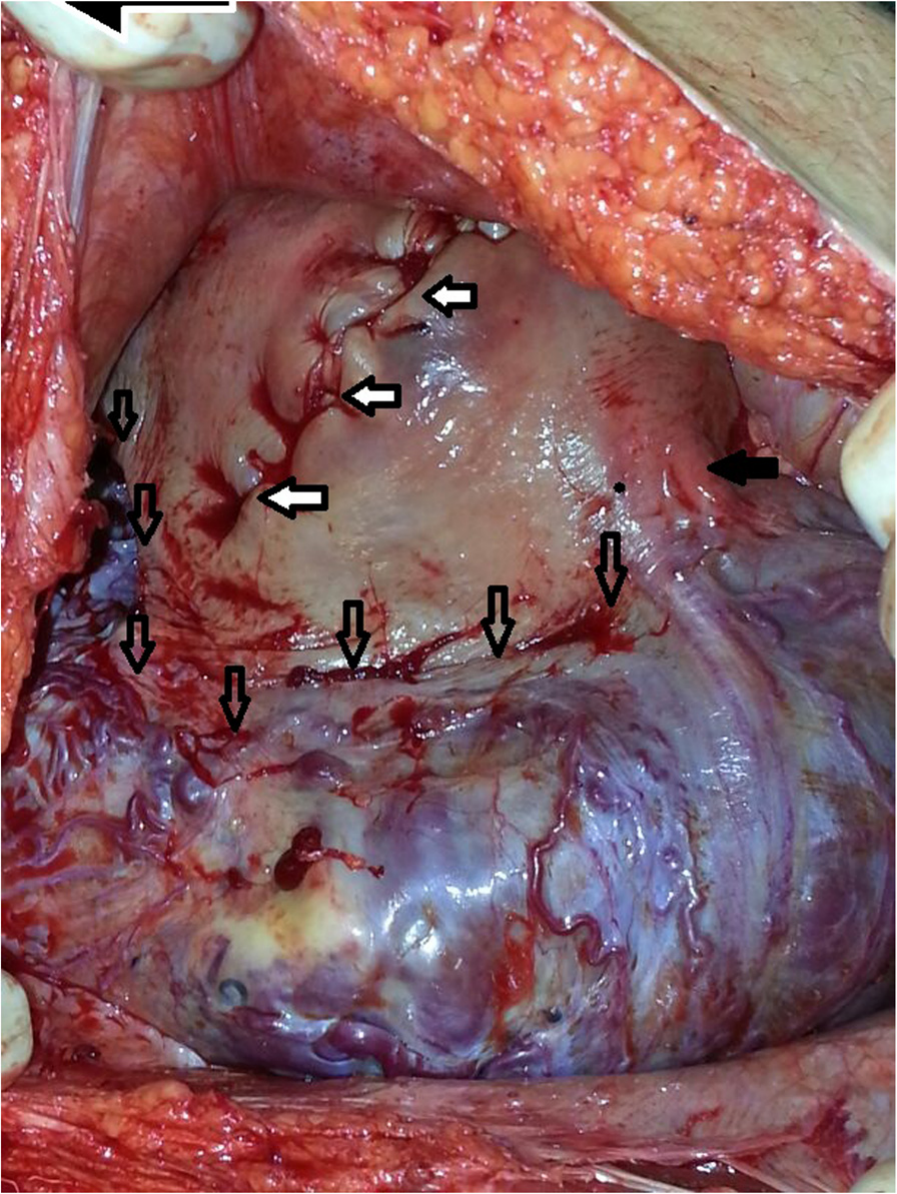
Uterus after the reparation (Intraoperative view). Anterior part of the repaired uterine incision could be seen (*white arrows*). Incision continues superiorly and posteriorly (not shown). Upper borders of the DC placentas are prominent (*transparent arrows*). *Black arrow* indicates the left round ligament

**Fig. 3:**
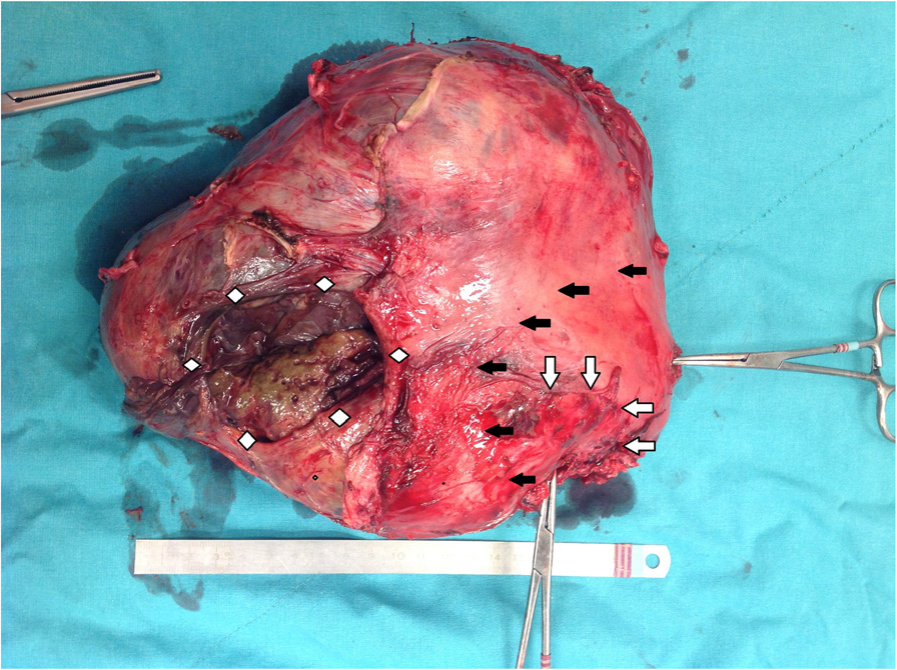
Uterus, removed. Total size of the uterus is 20 cm in length. Both round ligaments are seen clamped. Scar of the previous uterine closure is seen between the clamps (*white arrows*). Border of the lower uterine segment and uterine corpus (*black arrows*). Grayish–yellow placental tissue is seen through the area of uterine perforation (*white squares*)

CS was performed through a vertical midline abdominal incision. The incision was extended superiorly and inferiorly from the umbilicus in order to provide a major route to deliver fetuses without damaging the decidual-placental interface. Profuse, engorged, whorl-like patterned uteroplacental vessels were seen at the intraoperative evaluation at both parametra, particularly at the left side, and over the bladder (Fig. 2). The lower uterine segment and corpus uteri were both invaded by the placentas. Therefore, it was a necessity to perform a fundal incision rather than a classical incision to the uterus. Each of the umbilical cords was tied for twice with no. 1 silk sutures after delivery of fetuses. Both of the placentas were abandoned in situ. Myometrium was sutured primarily with no. 1 vicryl sutures in two layers (Fig. 2). Patient was not administered uterotonics during and after the procedure. Cefazolin was continued during post-operative period for 4 days. Intramuscular methotrexate was administered in 50 mg/m^2^ dose to enhance placental involution at the postoperative day 1. Patient was discharged at the postoperative day 4. She was advised for regular visits for once in two weeks. Transabdominal and suprapubic ultrasonographic survey, serum quantitative measures for leukocytosis and C-reactive protein (CRP) were conducted at every visit. At post-operative first week, ultrasonography yielded a 70 × 101 mm residual placenta at the left lower segment of the uterine cavity, and the second placenta which is 57 × 99 mm in dimensions at the right lower segment of the uterine cavity. Patient did not encounter any sort of bleeding. Serum CRP was negative (<0.5 mg/dL), and serum leukocyte counts were normal. Serum beta-hCG value was 324.86 IU/ml. The subsequent calls during follow-up did not yield any symptoms and abnormal test results until post-operative 5^th^ week. Serum CRP was measured 2.8 mg/dL at that time. She was administered oral metronidazole twice a day and 3^rd^ generation cephalosporins once a day for the treatment of the infection. The ultrasonographic measurement yielded shrinkage in dimensions of both placentas. One week later, she admitted to our clinic with severe inguinal and lower abdominal pain, and leukorrhea. There was a discomfort on abdominal palpation. Additionally, tenderness was prominent at inguinal regions, particularly at the left side. Maximum body temperature was 38.7 °C. Serum CRP was measured to increase 29.8 mg/dL. The second operation was planned. The operation was performed through the infraumbilical vertical midline incision. Uterus was pink to grayish in color. Distal part of the uterine corpus and bilateral adnexa were enlarged extremely due to uterine vasculature and mass of the placentas. Vesicouterine pouch was obliterated. Prophylactic ligation of bilateral hypogastric arteries was followed by routine technique for total hysterectomy (Fig. 3). The division of uterine neovasculature at the boundary of lower uterine segment and bladder and dissection of vesicouterine space was accomplished by using electrosurgical vessel sealing equipment. Our measures yielded a 1100 ml total blood loss at intraoperative period. Three units of erythrocyte suspension and 2 units of fresh frozen plasma were delivered to the patient at the perioperative and postoperative period. She was administered 3^rd^ generation cephalosporin for 7 days. Postoperative follow-up was uneventful. The patient was discharged at the postoperative 7^th^ day.

### Histopathological findings

Placental sectioning showed extensive hemorrhage in the villous tissue with extensive inflammatory response and tissue necrosis at the microscopy.

## Conclusions

The incidence of morbidly adherent placenta (MAP) increased from one in 30.000 live births in 1930 to one in 533 live births recently [2]. Causal factor is obvious endometrial defect related to previous surgeries, dilatation and curettages, previous placenta previa, advanced maternal age, multiparity, Asherman’s syndrome, and submucous leiomyoma [3]. Primarily, the presence of placenta previa together with afore mentioned factors should raise physician’s notice, since antenatal diagnosis of MAP and planning of the delivery could help to reduce morbidity and mortality [4]. Obstetric magnetic resonance imaging (MRI) is a superior and a feasible diagnostic method in situations where the exact diagnosis could not be reached by sonography [5]. In this case we did not perform MRI, because ultrasonographic features were vigorously suggestive of MAP. The timing for delivery could reasonably be postponed until 34 to 36 weeks, except for cases with massive vaginal bleeding and suspicion of extreme overgrowth to the adjacent organs.

Most of the cases of twin pregnancies with placenta percreta that were published so far reported uterine rupture at early gestational weeks of pregnancy [6–8]. Our case is interesting as it did reach to 35^th^ gestational week despite presence of increased placental burden of a twin pregnancy. Meanwhile, according to our best knowledge, there is no other case in the literature that both placentas were morbidly adherent and were remained in situ during the CS.

Detaching or making incision through adhesive placenta gives rise to massive blood loss, and complicates further steps of the surgery. Considering the prevention of hemorrhage, we preferentially performed a fundal rather than a classical incision to the uterus following a midline vertical incision to the skin. Therefore, as the first step of the treatment of MAP, we avoided even minor detachment of the placenta.

The following step in the optimal treatment usually addresses CH as the standard of care for MAP [2]. After the fetus is delivered the uterus is just taken out while the placenta is still attached. This approach is widely resumed as having the best outcomes. As an alternative approach, here, we abandoned the placenta in situ instead of performing hysterectomy [3]. Patient’s age, patient’s desire to preserve her uterus, our belief to facilitate surgical outcomes and to decrease need for massive transfusion were the reasons to perform this type of surgery.

In patients with retained placenta, the concern of ‘what to do with the placenta’ arises. As it is theorized that placenta brakes down in time and would be pulled out partially, one can wait for the signs of expulsion of the placenta. Although the thought is reasonable, the journey to the summit is very long and troublesome. Regarding that theory, there are case series reporting favorable outcomes in patients with MAP in singleton pregnancies [1, 9]. In this case, placenta did not brake down in a long period of time. Moreover, it caused metritis. Therefore, presence of uterine infection necessitated performing emergency surgery at a time before planned surgery for excision of the placental tissues together with metroplasty. Nevertheless, two of our main concerns were reached. Among them, first was to decrease the amount of transferred blood products, and decrease morbidity related to massive transfusion. The second was to decrease co-morbidities related to damaging the adjacent organs during emergency hysterectomy. The other and noble concern, which was to give a chance to preserve and recover the uterus, could not be reached. In such a circumstance, we advocate to ensure an exact control over demographics and medical condition of the patient, the extent of the invasion of placenta, the total volume or amount of the retained placenta, total uterine size comprising placental volume, and the hematoma in the uterine cavity together with the signs of cervical dilatation and expulsion of placenta.

Ligation (LHA) or obliteration (OHA) of the hypogastric arteries were reported to be ineffective if performed without hysterectomy to control major pelvic hemorrhage in up to 60 % of cases of MAP [4, 10, 11]. As it is known that bilateral LHA is a time consuming and ineffective procedure in patients with MAP, we did not intend to perform prophylactic LHA during CS in this case.

The data regarding the long-term reproductive outcomes after conservative treatment of patients with MAP are limited [1, 6, 12, 13]. One can assume that the physiology of the endometrium has not been corrected, and the theoretical risk of recurrent MAP rises in this population. However, we could improve implantation site within endometrial cavity by repairing the defective zone related to previous CSs owing to a popular theory of implantation of the embryo directly on the endomyometrial junction [14]. Despite this blurred picture and the increased risk of recurrent MAP, a chance to conceive should be considered in meticulously selected cases such as very young parturients.

In conclusion, leaving the placenta in situ seems to be a logical alternative to CH in patients with MAP. However, the surgeon should be aware of infectious, hemorrhagic, and psychological complications related to retained placenta. In presence of MAP with DC pregnancy, it appears to increase risk of maternal infection, and increase maternal morbidity. Therefore, we advocate the idea that uterine conservation approach should be personalized with meticulous patient selection [15].

## Consent

Written informed consent was obtained from the patient for publication of this Case report and any accompanying images. A copy of the written consent is available for review by the Editor of this journal.

## Abbreviations

CRP: C-reactive protein
CS: Cesarean section
DC: Dichorionic
LHA: Ligation of the hypogastric arteries
MAP: Morbidly adherent placenta
MRI: Magnetic resonance imaging
OHA: Obliteration of the hypogastric arteries
